# Sorption of Eu(III) on Eibenstock granite studied by µTRLFS: A novel spatially-resolved luminescence-spectroscopic technique

**DOI:** 10.1038/s41598-019-42664-2

**Published:** 2019-04-18

**Authors:** K. Molodtsov, S. Schymura, J. Rothe, K. Dardenne, M. Schmidt

**Affiliations:** 1 Helmholtz-Zentrum Dresden-Rossendorf, Institute of Resource Ecology, Dresden, Germany; 2 Karlsruhe Institute of Technology (KIT), Institute for Nuclear Waste Disposal (INE), Karlsruhe, Germany

**Keywords:** Geochemistry, Characterization and analytical techniques, Geochemistry

## Abstract

In this study a novel technique, micro-focus time-resolved laser-induced luminescence spectroscopy (µTRLFS) is presented to investigate heterogeneous systems like granite (mainly consisting of quartz, feldspar, and mica), regarding their sorption behavior. µTRLFS is a spatially-resolved upgrade of conventional TRLFS, which allows point-by-point analysis of single minerals by reducing the beam size of the analytic laser beam to below the size of mineral grains. This provides visualization of sorption capacity as well as speciation of a luminescent probe, here Eu^3+^. A thin-section of granitic rock from Eibenstock, Saxony, Germany was analyzed regarding its mineralogy with microprobe X-ray fluorescence (µXRF) and electron probe microanalysis (EPMA). Afterwards, it was reacted with 5.0 × 10^−5^ mol/L Eu^3+^ at pH 8.0 and uptake was quantified by autoradiography. Finally, the µTRLFS studies were conducted. The results clearly show that the materials interact differently with Eu^3+^, and often even on one mineral grain different speciations can be found. Alkali-feldspar shows very high uptake, with an inhomogeneous distribution, and intermediate sorption strength. On quartz uptake is almost 10-fold lower, while the complexation strength is higher than on feldspar. This may be indicative of adsorption only at surface defect sites, in accordance with low hydration of the observed species.

## Introduction

To develop a safety concept for an underground nuclear waste repository, it is essential to understand the interaction processes between radionuclides and their environment^1^. In the event of water intrusion into the containment, interactions with solid phases, e.g. sorption and incorporation processes, play the main role as retention mechanisms, which prevent radionuclides from entering the biosphere. In the case of the host rock as the last barrier the repository is embedded in, these solid phases are mostly heterogeneous, complex systems like granite or clay consisting of a mix of various minerals^2^. In Germany, both systems as well as salt rock are possible candidates to use as a host rock^3^, while in other countries like Scandinavia, Russia or China granite is favored^4–6^. In this study the sorption behavior of Eu(III) on granite is investigated, which serves as an chemical homologue for trivalent actinides Am(III), Cm(III), and Pu(III). Am and Pu are the dominant contributors to the radiotoxicity of spent nuclear fuel after about 100 years after removal from the reactor^7^. Am(III) is always trivalent in aqueous solution and also Pu is expected to be present in its trivalent state under the reducing condition prevailing in deep geological formations^1^.

Most research on sorption speciation of trivalent lanthanides and actinides is done as batch or spectroscopic studies with single and isolated constituents of granite which are mainly quartz (SiO_2_), feldspar (alkali-feldspar: (K/Na)[AlSi_3_O_8_]; plagioclase: (Ca/Na)[AlSi_2/3_O_8_), which is a family of tectosilicates with embedded K^+^, Na^+^, and Ca^2+^, and mica (muscovite: KAl_2_(F,OH)_2_[AlSi_3_O_10_]; biotite: K(Fe_3_/Mg_3_)(F,OH)_2_[AlSi_3_O_10_]), phyllosilicates with a layered structure and K^+^ in the interlayer^2^. The sorption of Eu^3+^ on those mineral components has been studied previously.

For Eu^3+^ sorption on quartz a two-step sorption mechanism is reported. The first sorption complex begins to form at pH 4.5 as a bidentate surface complex, which starts to hydrolyze to a second species at a pH value of 6.0, reaching its maximum at pH 7.5^8,9^. Both sorption species are inner-sphere complexes with five water/OH^−^ molecules left in the first hydration shell. In addition, the metal ions can form complexes with dissolved silica on the quartz surface. The reaction starts at pH 6.0 and at pH 9.0 the amount of water molecules left is reduced from five to two^10^. Over time, this reaction may lead to the incorporation of Eu^3+^ into silica, often in the form of Eu^3+^-bearing colloidal silica. On alkali-feldspar a similar sorption behavior was reported with inner-sphere sorption starting at pH 4.5 (albite, Na-feldspar) or 5.5 (orthoclase, K-feldspar)^11,12^. At a pH of 6.0 the formation of a second species is observed on both feldspar end members, which was interpreted as hydrolysis of the first inner sphere sorption species. A similar dissolution/complexation mechanism as described above for quartz has not been observed on feldspars.

The sorption behavior of mica is different for biotite and muscovite. Biotite is mainly accumulating trivalent radionuclides in the interlayer by exchanging them for K^+^. It remains unclear whether this occurs as inner or outer sphere complexes^13^. This sorption process starts at pH 2.5 and reaches its maximum at pH 3.5. Ion exchange with Na^+^ or other cations acts as a competitive process and therefore the sorption process is dependent on the ionic strength. Muscovite does not show this interlayer accumulation^14^. Sorption of Eu^3+^ on Muscovite starts at pH 3.0 and increases until pH 8.0. Up to pH 7.0 higher ionic strength is found to reduce uptake of Eu^3+^ ^15^. This is due to the fact that mostly outer-sphere sorption occurs on the surface of muscovite at pH 5.0 and below. With rising pH, inner-sphere complexes form. At pH 7.0 these complexes are identified as mono-dentate hydroxide complexes and at pH 9.0 as mono-dentate carbonate complexes. With the rare earth ion homologue Y^3+^, the co-existence of inner sphere, outer sphere, and extended outer sphere complexes was observed on the muscovite mica basal plane at pH = 5.5^16^.

Predicting the behavior of the whole system based on this information is difficult. On the one hand, although understanding sorption processes on the single constituents of granite helps understanding the sorption behavior of the whole system, it cannot serve to fully understand the real system and even the sum of those studies can only act as a model system for granite. For example, studying isolated minerals cannot reveal how the minerals influence sorption of Eu^3+^ on the other phases. In addition, real granite shows heterogeneities that cannot be studied or averaged like grain boundaries, diverse crystal orientation, localized mineral alteration and inclusion of minor minerals, which are usually not subject of research^17–20^. On the other hand, batch studies of the whole system can only describe sorption behavior as an average and the relationship between mineral phase and Eu^3+^ species is lost. The same problem arises for bulk spectroscopic studies. It should also be taken into account that minerals for single mineral studies could have grown under different geological conditions than those in granite and that many batch studies use minerals altered by milling, which will create surface topologies different from natural fractures. An effect that may be more pronounced for some minerals, e.g. soft micas with a preferential cleavage direction may be altered more strongly than harder quartz.

To understand how the described heterogeneities influence the sorption behavior of granite, it is necessary to study the real system directly, maintaining the species-mineral relationship, which can only be achieved by spatially-resolved investigations. However, most spatially-resolved studies of radionuclide migration focus on the quantitative distribution of radionuclides and their analogues^21^, often resorting to co-location with other elements to obtain further information^13,22–24^. If the speciation of the adsorbate cannot be determined in the spatially-resolved experiment directly, one must rely on correlation with results from single phase studies to deduce the mineral-species relationship. Studies of Eu^3+^ sorption on granite with electron probe micro analysis (EPMA) and laser-ablation ICP-MS (LA-ICP-MS)^13,21^ at low to neutral pH show a heterogeneous distribution, mostly on biotite, which is affected by its topology and alteration during the sorption process. There is also an accumulation of the metal ions at grain boundaries between quartz and feldspar^13^. Even though the accumulation process on biotite is described, this type of studies cannot reveal sorption speciation. Without that information, it is not possible to predict sorption behavior at other environmental conditions than the ones that were chosen to conduct these studies. An approach to get chemical information of the sorption species makes use of fluorescence lifetime of Eu^3+^ as a fingerprint^25^. By this approach it was shown, that sorption on all major components of granite and on granite itself is heterogeneous. On feldspar multiple species could be identified within a wide range of lifetimes. These can be separated into two regimes, one regime of inner-sphere species with lifetimes above 110 µs and one regime of unidentifiable species with lifetimes below 110 µs. On biotite the only detectable species was the Eu^3+^ aquoion. These lifetime fingerprints of feldspar and biotite were also found on granite. Nevertheless, lifetime measurements only determine the presence of quenchers in Eu^3+^’s coordination shell, and can only differentiate changes in the hydration shell of species, and only if no other quenchers, e.g. Fe or Mn, are present. More information about speciation would have to be obtained from the full luminescence spectra.

To combine the excellent speciation capabilities of Eu^3+^ time-resolved laser-induced fluorescence spectroscopy (TRLFS) (see below) with the spatial resolution of microprobe techniques, we have developed µTRLFS, a spatially-resolved upgrade of conventional TRLFS. For µTRLFS, a laser beam is focused to a size of ~30 µm, of the same order of magnitude as mineral grain sizes in natural crystalline rock. By scanning a sample through the focus spot, it is possible to reconstruct maps of the spectral information. In the case of Eu(III), the speciation can be derived from this spectral information, due to its emission bands properties^10,15,26,27^. Typically, two bands are used to analyze the coordination environment of the Eu^3+^ luminescent probe, the (^5^D_0_ → ^7^F_1_) transition (^7^F_1_ for short) as a magnetic dipole transition is by first approximation independent of Eu^3+^’s coordination sphere. It typically serves as an internal reference. The (^5^D_0_ → ^7^F_2_) transition (^7^F_2_ for short) is an electric dipole transition and shows the so-called “hyper-sensitive effect”, a strong increase in relative intensity with increasing coordination strength^28^. The intensity ratio of these two transitions ^7^F_2_/^7^F_1_ can then be used as a measure of sorption strength. This speciation information is in addition to the whole luminescence intensity, which is correlated to the sorption capacity, much like in other microprobe techniques, e.g. µXRF. A third source of information is the previously discussed time dependency of the luminescence signal. Because the water molecules of the first hydration shell of Eu^3+^ quench its luminescence, it is possible to calculate the amount of water molecules remaining in the coordination sphere from the luminescence lifetime using the equation of Horrocks (see below)^29^.

Consequently, µTRLFS allows characterizing the speciation of Eu^3+^ (as well as other luminescent probes) in a single spot of ~30 µm. This is possible due to the trace concentration sensitivity of the method, due to the high luminescence intensity of Eu^3+^. The selection of excitation wavelength, time windows, and spectral detection range also means that measurements show very low background, and luminescence intensity can be assigned to Eu^3+^ unambiguously. While the spatial quantification of sorption processes is possible with many methods, point-by-point speciation is a major advantage of µTRLFS.

Here, we demonstrate the first application of our µTRLFS setup to elucidate the sorption species distribution of Eu^3+^ on a natural granite from Eibenstock, Germany. Sorption of Eu^3+^ was first characterized in batch sorption studies, and a careful characterization of the materials mineral composition was performed. Finally, Eu^3+^ speciation on the crystalline rock was characterized on the molecular level by µTRLFS under conditions where Eu^3+^ sorption is at its maximum, but dissolution of the substrate and precipitation of Eu^3+^ solids are not yet prevalent ([Eu^3+^] = 5.0 × 10^−5^ mol L^−1^, pH = 8.0, 0.1 M NaCl as background electrolyte, in air). These conditions are reasonably close to observed pH values and ionic strengths of ground water in several granitic formations^30–33^.

## Results

### Batch Experiments

**Figure 1:**
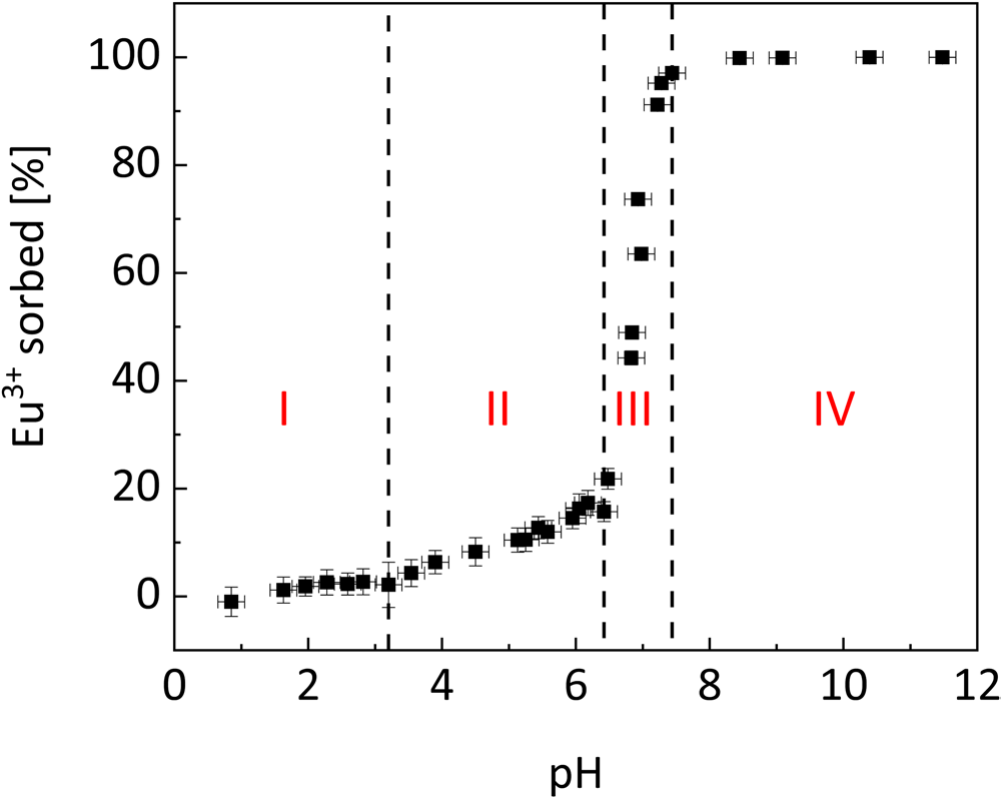
Batch data of sorption experiment with 5 × 10^–5^ mol L^−1^ Eu^3+^ in 0.1 M NaCl on ground Eibenstock granite (S/L = 2 g/L). The four indicated zones are intended as a guide-to-the-eyes, for interpretation of the results.

Batch experiments show the quantitative sorption behavior of Eibenstock granite (Fig. 1), which can be classified into four different regimes. In the most acidic pH range from 0.8 to 3.2 (**I**) no Eu^3+^ sorption occurs within the errors of the measurement. Between pH 3.2 and 6.4 (**II**) Eu^3+^ sorption increases gradually from <2% to ~20% and then (**III**) increases rapidly to 100% sorption at pH 7.4. At even higher pH Eu^3+^ sorption remains constant at 100% (**IV**), and no desorption can be observed.

We can attempt to interpret this multi-step sorption behavior based on the sorption properties of Eibenstock granite’s mineral components. Due to their permanent negative surface charge^34,35^, the mica components would be expected to be most reactive at acidic pH > 2.5 (**II**), likely outer sphere (OS) complexes are formed at first in the case of muscovite^15^. In the case of biotite the dependency of ionic strength on the sorption capacity indicates the formation of outer-sphere complexes too^13^. As the mica content in Eibenstock granite (11.5%) is much lower than quartz (45%) and feldspar (42.5%), the fraction of sorption on that mineral should be low, especially as the high background electrolyte concentration should further suppress the formation of OS complexes^13,15^. With increasing pH (pH > 4.5) also the formation of inner sphere (IS) surface complexes on quartz^9,10^ and feldspar^11^ is expected. Regime **III** is most likely dominated by sorption on feldspar, where very similar sorption edges at pH ~ 6.5–7.5 have been observed for Eu^3+^ previously^12^ and sorption on quartz occurs slightly shifted to lower pH between pH 6.0 and 7.0^36^. Again it is not possible to distinguish potential contributions from micas and quartz. In regime **IV** sorption remains constant at 100%, which would be expected for Eu^3+^ sorption on any of the major components. Surface precipitates may also play a role at such high pH values, e.g. Eu(OH)_3_ or Eu(OH)CO_3_^37^. In summary, batch experiments are helpful in describing Eu^3+^ sorption quantitatively, but fall short of describing the sorption process with regard to sorption modes or involved mineral phases. Spatially-resolved spectroscopy measurements shall help revealing the speciation on each mineral at pH 8.0 in regime **IV**, where sorption is at its maximum, but surface precipitation is still of minor importance. In order to be able to derive species – mineral associations from the spatially-resolved spectroscopic data, first the mineral phase distribution in the ROI must be characterized.

### Mineral phase distribution

From µXRF and electron microprobe measurements, the elemental distribution on the sample surface can be determined. The combination of both methods helps identifying the mineral distribution not only directly at the surface, but also reveals what minerals are directly underneath, as the two methods have slightly different detection depths. The detection depth of EPMA is dependent on the penetration depth of the incident electron beam, which is approximately 2–3 µm. For µXRF, the detection depth is dependent on the penetration depth of the generated fluorescence photons, because the incident photon beam of 18 keV has a higher penetration depth than the fluorescence that needs to escape the penetrated material again to the detector. Due to that the detection depth of µXRF is element specific and is estimated to be as low as ~7 µm for K and reach >400 µm for Rb. This estimation is based on the mass attenuation law for photons by using the mass attenuation coefficients of the main constituents of granite^38^. In contrast to µXRF, EPMA is also capable of measuring elements with atomic numbers below Ar. Along with its higher spatial and also spectral resolution this is useful to definitely identify all of the minerals at the surface, because some of the major constituents of granite consist of elements mainly or even only with atomic numbers below that of Ar and can therefore not clearly be identified by µXRF. Both methods are principally suited to locally quantify the Eu^3+^ distribution, but were ultimately unsuccessful in this particular case (see below). Thin-section microscopy is a helpful addition for the identification of mineral grains in the sample.

**Figure 2:**
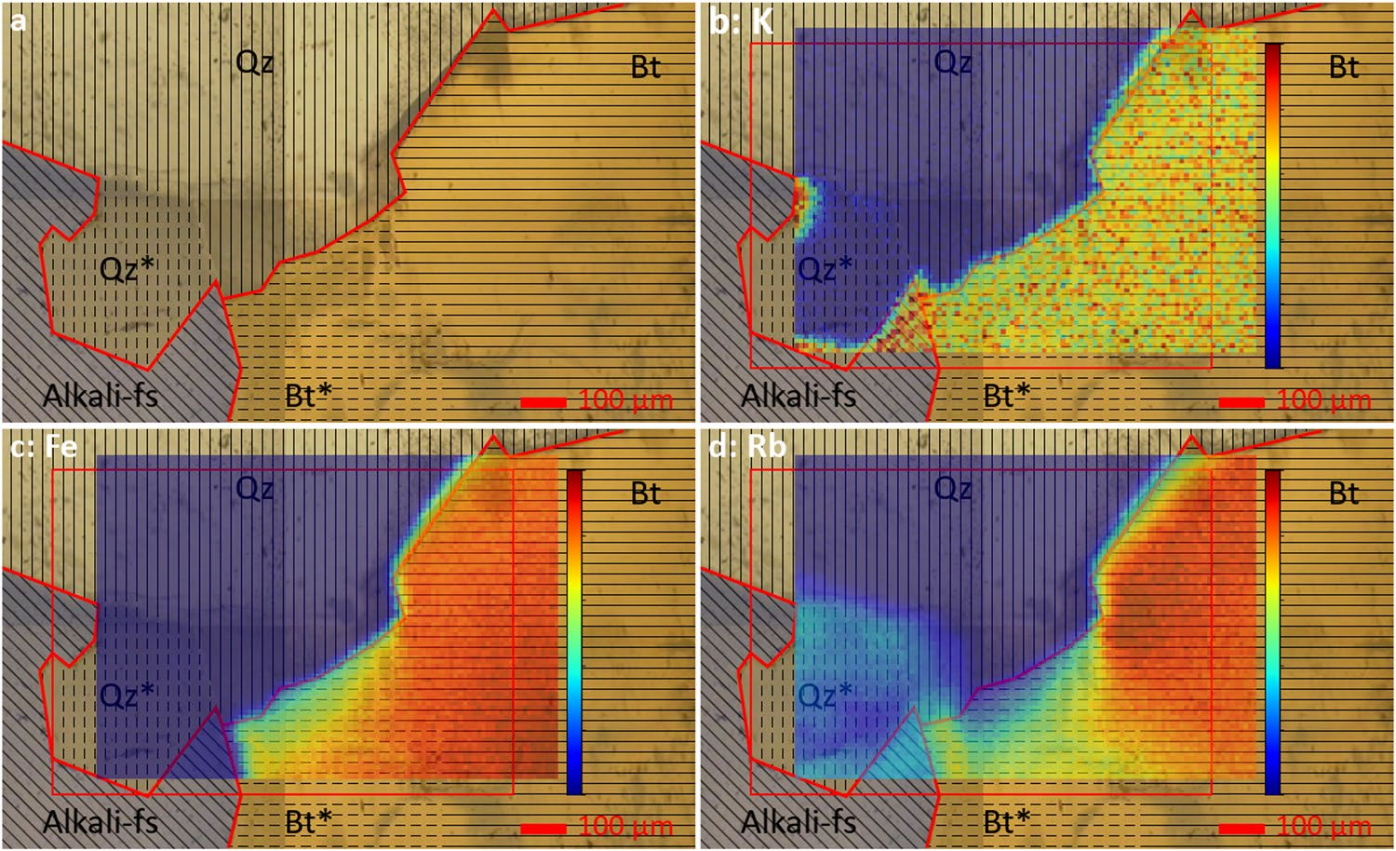
Colorized mineral distribution of the ROI (**a**) with quartz (Qz), quartz layer over alkali-feldspar (Qz*), alkali-feldspar (Alkali-fs), biotite (Bt) and biotite layer over quartz (Bt*) along with µXRF results for potassium (**b**), iron (**c**) and rubidium (**d**) whereas the red outlines indicate the grain boundaries at the surface of the sample and the red rectangle indicates the ROI used for µTRLFS measurements.

Figure 2 shows the mineral distribution determined by µXRF, EPMA and thin-section microscopy along with the elemental distributions measured by µXRF to visualize the differences between Bt and Bt* and between Qz and Qz*. The ROI consists of three minerals: alkali-feldspar, quartz, and biotite. Alkali-feldspar (Alkali-fs) is mostly K-feldspar with small amounts of Ca, mixed lamellarly with pure Na-feldspar. Biotite (Bt) is annite-like with 3–4 times higher Fe content than Mg. In both biotite and feldspar, K^+^ has been partially substituted by Rb^+^. The grain boundary between these minerals is enriched in Al^3+^ and Mg^2+^ in comparison to the minerals themselves. Other grain boundaries do not show any divergent content of elements relative to the adjacent minerals. Quartz (Qz) is not pure SiO_2_ but has some Mg^2+^ homogeneously incorporated, to roughly the same extent as Na-feldspar, while K-feldspar has an even higher Mg content.

The two areas labelled as Qz* and Bt* are regions where two minerals are layered on top of each other. The layering can be characterized by µXRF (Fig. 2), due to its energy dependent sampling depth. In the area labelled Qz* no K fluorescence signal is detectable, however, we do observe Rb fluorescence. Likely, the mineral below quartz contains both K^+^ and Rb^+^, but only Rb fluorescence is detected, due to its significantly higher energy (Rb *Kα*_1_ = 13,4 keV, *Kβ*_1_ = 14,96 keV) relative to the K K-shell-transitions (*Kα*_1_ = 3,31 keV, *Kβ*_1_ = 3,59 keV). Because there is no detectable Fe fluorescence in this area, Qz* must be a quartz layer on top of Alkali-feldspar. In the Bt* region the K signal is homogeneously spread, but with rising energy of the fluorescence signal (${E}_{Rb} > {E}_{Fe} > {E}_{K}$), the intensity gets lower. Thin-section microscopy reveals that the underlying mineral is quartz.

### Local Eu^3+^ speciation (µTRLFS)

The µTRLFS measurements yield maps of luminescence intensities and peak intensity ratios, as well as luminescence lifetimes for selected spots. The area investigated was the same ROI, for which the mineral composition was determined by µXRF and EPMA.

**Figure 3:**
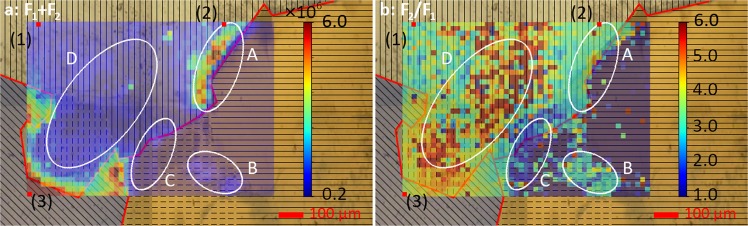
Map of total intensity of ^7^F_1_ and ^7^F_2_ emission bands (**a**) and map of ratio of ^7^F_2_/^7^F_1_ emission bands (**b**) with tagged regimes A–D and lifetimes (1–3) (red dots).

In Fig. 3, the maps of luminescence intensity (a) and ^7^F_2_/^7^F_1_ peak ratio (b) is shown. Luminescence intensities vary by a factor of ~20 over the whole map, with the strongest luminescence on Alkali-fs, and lowest values for Bt. We also observe a wide range of ^7^F_2_/^7^F_1_ ratios, from ~1 to almost 6. Here, highest peak ratios are found on Qz, with lowest peak ratios again on Bt. Both, intensity and peak ratio show distinct areas of high or low intensity, which are related to the mineral grains, but not identical with them. This can for example be seen in the region marked **B** on Bt* which has a higher uptake of Eu^3+^ than Bt or Bt*, or the grain boundary between Qz and Bt marked **A**, where uptake is higher than on Qz and Bt, but the peak ratio is smaller than on Qz in region **D**.

The highest total fluorescence signal was detected on Alkali-fs, in particular in the lower-left corner of the ROI, which exhibits ~10× higher luminescence than other alkali-feldspar regions. The ^7^F_2_/^7^F_1_ peak ratio is homogeneous over the whole mineral grain at ^7^F_2_/^7^F_1_ ≈ 4.0. This indicates two properties of Eu^3+^ sorption on feldspar: 1. sorption is relatively strong, with a peak ratio indicating mostly inner sphere sorption^26,39^, and 2. the differences in sorption capacity are related to available sorption sites but not differences in sorption strength.

On quartz, the luminescence intensity is distributed more homogeneously. The luminescence intensity on Qz is significantly lower than on Alkali-fs, indicating preferential Eu^3+^ sorption on feldspar. When considering the ^7^F_2_/^7^F_1_ peak ratio, however, it is noticeable, especially for region **D**, that the peak ratios are actually the highest within the ROI, with values up to ^7^F_2_/^7^F_1_ = 6.0. A notable exception from the homogeneous distribution is area **A**, i.e. the grain boundary between Qz and Bt. At this boundary more Eu^3+^ is accumulated than on Qz and Bt themselves and the signal strength is of the same order of magnitude as on Alkali-fs. In contrast, the ^7^F_2_/^7^F_1_ peak ratio in **A** is lower than in **D**, once again similar to Alkali-fs with values ^7^F_2_/^7^F_1_ ≈ 3.0–4.0. The grain boundary between Qz and Bt* at area **C** does not show a similar behavior, but rather appears similar to Bt* and Bt, even in the parts that are located on Qz.

Biotite itself shows very low luminescence except for area B on Bt*. Due to the low signal to noise ratio the peak ratio could not be evaluated. **B** exhibits the strongest luminescence on Bt and Bt*, which is, however, still lower than on quartz. The peak ratio in **B** is around 3.0, slightly lower than on Alkali-fs and much lower than on Qz in area **D**. The grain boundaries between quartz and feldspar and feldspar and biotite do not noticeably deviate from the behavior of the bulk mineral grains.

Due to the data analysis routine available at the time of measurement, points for lifetime measurements had to be selected before points of interest could be identified from the maps in Fig. 3. In the following, we will discuss representative points on Qz (1) and Alkali-fs (2), as well as the Qz/Bt grain boundary (3). The signal strength on Bt and Bt* was too low for a reliable determination of luminescence lifetimes, even in region **B** with higher intensity. All lifetimes are summarized in Table 1.

**Table 1:** Determined lifetimes of selected points for the main components of Eibenstock granite.

Spot	Mineral	Lifetimes [µs]	N(H_2_O) ± 0.5
(1)	Quartz	72 ± 5	—
		350 ± 40	2.4
(2)	Alkali-fs	140 ± 8	7.0
		1100 ± 60	0.4
(3)	Qz/Bt grain bound.	110 ± 8	9.0
		1470 ± 700	−0.8

On Qz, we observe a biexponential decay behavior, which can be fit with a short lifetime of 72 ± 5 µs and a longer lifetime of 350 ± 40 µs. The longer lifetime corresponds to 2.4 H_2_O quenchers in Eu^3+^’s first coordination sphere, according to Eq. 3. This is somewhat lower than the typical values for inner sphere complexes where 4–5 H_2_O remain with the adsorbed ion. The release of additional water molecules from Eu^3+^’s hydration sphere may indicate sorption in a surface defect site with a higher coordination number. The short lifetime on the other hand is shorter than the reference value found for the Eu^3+^ aquo ion (110 µs^40^). This can be caused by hydrolysis of Eu^3+^ surface complexes or the presence of transition metal centers, particularly Fe and Mn, with suitable acceptor electron energy levels in close vicinity to Eu^3+^ ^40–42^. Our elemental maps of this region do not indicate the presence of Fe or other transition metal quenchers around (1), so we tentatively assign the short lifetime to hydrolyzed surface complexes.

Similarly, we observe two lifetimes on Alkali-fs (2), a short lifetime of 140 ± 8 µs and a longer lifetime of 1100 ± 60 µs. The short lifetime corresponds to seven water molecules remaining in the first coordination sphere of Eu^3+^, indicating an inner sphere complex with weak attachment to the surface. The long lifetime corresponds to near-complete loss of hydration with 0.4 H_2_O remaining with the adsorbed Eu^3+^ ion, which would indicate incorporation, or a surface incorporation process^43^.

Lifetime analysis in region A yields one lifetime of 110 ± 8 µs corresponding to the Eu^3+^ aquo ion, and a long, but poorly defined, lifetime of 1470 ± 700 µs, representing a range of 0.0–0.8 H_2_O quenchers around Eu^3+^. The large error limits the reliability of this value, however. The major sorption mode in this area is then outer sphere sorption. Generally, the lifetimes on all mineral phases do not suggest the formation of Eu^3+^ solids or particles^44,45^.

### Quantitative Eu^3+^ distribution (µXRF, EPMA, Autoradiography)

The quantification of Eu^3+^ uptake on the interface is principally also possible with other techniques than µTRLFS, such as µXRF and EPMA, which were used for mineral phase identification in this study. In this particular system, however, both approaches were unsuccessful. Detailed discussions of the problems we encountered can be found in the electronic annex (EA). Briefly, µXRF suffered from X-ray fluorescence line overlap with Mn, which is a common trace element in granitic rocks^46,47^. The K_α_ line of Mn and L_α_ line of Eu overlap, and other edges were not accessible in our experiments. A better energy resolution circumvents this problem for EPMA, but the method was not sensitive to the very small amount of Eu^3+^ that is adsorbed on the material. In our experiments no significant Eu signals could be detected.

Our attempts to independently quantify Eu^3+^ uptake were more successful using autoradiography with ^152^Eu. Unfortunately, autoradiography was performed after μTRLFS experiments, and revealed additional topography on the surface, likely caused by laser ablation, and led to higher sorption in the mapped area than outside of it (see EA). It should be noted that repeated μTRLFS measurements in the same spot produced spectra with very good reproducibility, indicating that the topographical changes occur on a small scale and do not significantly affect adsorbed Eu^3+^ until the sample is re-submerged in Eu^3+^ containing solution.

**Figure 4:**
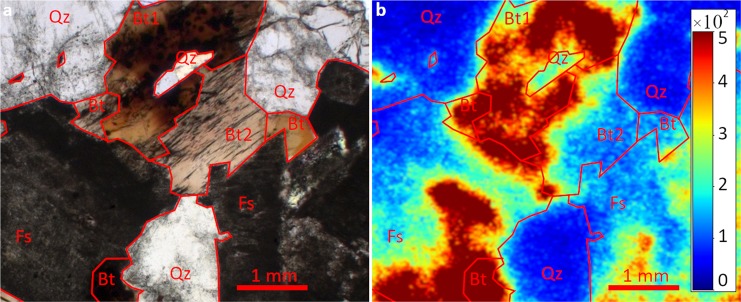
Autoradiography image of a larger region on the same sample with underlying image of thin-section microscopy (**a**) and standalone (**b**) with red outlines of each mineral phase at the surface (Qz = quartz, Fs = feldspar, Bt1/2 = biotite).

Initial experiments (see EA) had demonstrated that the spatial resolution of the autoradiography maps is not comparable to μTRLFS. Thus, we performed additional autoradiography experiments on the same sample on a larger area outside of the μTRLFS ROI, to confirm that autoradiography finds the same general trend, regarding sorption quantity on the various mineral components. The results are shown in Fig. 4.

The autoradiography map confirms the trends observed with μTRLFS: Eu^3+^ sorption is significantly higher (3–10×) on feldspar than on quartz, it also shows that biotite is intermediate between the two other minerals. Quartz areas (Qz) show the lowest intensity (<200 cts) with a homogeneous distribution. The biotite in this ROI can be divided into two areas, Bt1 and Bt2. Bt1 has a high intensity between 1000 and 2000 counts, which is distributed relatively heterogeneous, while sorption on Bt2 is more homogeneous but of lower quantity with intensities of around 500 cts. In both cases, accumulation of Eu^3+^ on biotite grain boundaries is noticeable. On feldspar (Fs) sorption occurs more heterogeneously with intensities ranging from 500 up to 2000 cts. The same relative behavior of the three major components is observed over the whole sample, with few hot spots with intensities of up to 40000 counts.

## Discussion

The first results from spatially-resolved time resolved laser luminescence spectroscopy (μTRLFS) reveal the sorption behavior of Eu^3+^ on a natural granite sample from Eibenstock, Germany. The results highlight the differences between the mineral components in both sorption capacity and strength of the surface complexation. Moreover, results demonstrate that not only heterogeneities like grain boundaries show diverse sorption behavior in comparison to the adjoining minerals, but even within one mineral grain the sorption behavior varies.

Autoradiography qualitatively confirms the quantitative Eu^3+^ sorption measured with µTRLFS, and adds information in the case of biotite. Quartz has the least amount of adsorbed Eu^3+^, which is spread homogeneously, while feldspar shows a 5–10 × higher sorption capacity, but more heterogeneously distributed. Sorption on biotite is intermediate in quantity, higher than on quartz, but lower than on feldspar, and appears to depend greatly on grain orientation. A possible explanation may be found in the differing accessibility of interlayer spaces for preferred sorption. For both grains we observed preferential adsorption close to mineral grain boundaries of biotite.

The highest uptake was found on Alkali-fs, but both μTRLFS and autoradiography show that the sorption capacity is not homogeneous within a single mineral grain. The heterogeneity of the sorption on feldspar was not observed before and is not correlated to the occurrence of K-, Na-, and Ca-feldspar. The speciation of Eu^3+^ adsorbed on feldspar shows a moderate ^7^F_1_/^7^F_2_ ratio, indicative of IS complexation^26,39^. The lifetime measurement on feldspar shows a relatively strong hydration of the surface complexes, with seven water molecules bound to Eu^3+^, rather than the typical 4–5 water molecules bound to IS complexes. This analysis may be influenced by the presence of transition metal inclusions in the material, however, which could act as quenchers reducing the lifetime of the adsorbed species^41,42^. Another possible explanation is the formation of hydrolyzed surface complexes, which may exhibit shorter lifetimes^40^. The formation of such hydrolyzed surface species is in good agreement with earlier studies of the sorption behavior of trivalent lanthanides and actinides on feldspars^11,12^. The additional long lifetime close to complete loss of hydration, indicates incorporation of Eu^3+^. This is not an indication of an incorporation process, which would be unlikely in the short time frame of our experiments, but rather indicates the presence of Eu^3+^ as a natural trace contaminant in feldspar^48,49^.

Sorption of Eu^3+^ on Qz is both much lower and more homogeneous than observed for Alkali-fs, the only exception is the grain boundary between Qz and Bt, marked as area A in Fig. 3. When examining Eu^3+^’s crystal field as a measure for the speciation of Eu^3+^ on Qz, it becomes obvious that both very strong (^7^F_2_/^7^F_1_ > 6) and fairly weak bonding (^7^F_2_/^7^F_1_ < 3) occur, often on neighboring pixels in our mapping. Typically, strong surface bond strength would be expected to be associated to high uptake capacity. As this is clearly not the case on Qz in our experiment, we must assume that the number of available surface sites is severely limited. A possible explanation for this behavior could be that bonding of Eu^3+^ to Qz in a competitive situation including the other mineral phases, biotite and feldspar, is only possible on surface defect sites, such as kink or edge sites. This would simultaneously explain the strong crystal field exerted on Eu^3+^, as such sites would be expected to present environments with high coordination numbers. In that case sorption on Qz would only occur, where such surface sites are available, but not at all, or only as weakly associated OS complexes, on the rest of the Qz surface. A possible alternate explanation could be the formation of ternary surface complexes, e.g. with carbonate^50^. The release of silicate should be too low to have a significant impact at pH 8.0^51^. Ternary complexes can satisfactorily explain the strong ligand field observed in the ^7^F_2_/^7^F_1_ ratio distribution, but it is unclear why their distribution would vary so strongly from pixel to pixel. Lifetime measurements confirm the presence of a surface complex with a large number of replaced water molecules, either through multiple bonds to the surface, or through bonds to an additional ligand from the solution. A second shorter lifetime may once again indicate hydrolysis of the surface complexes.

The grain boundary between Qz and Bt (**A**) exhibits distinct sorption properties. Here, sorption is significantly higher than on either of the adjacent mineral grains, but the complexation strength (^7^F_2_/^7^F_1_ ~ 3) is lower than in the maxima on Qz. A molecular level interpretation is difficult, as obviously no reference data can exist for such a mineral grain boundary. The lifetimes indicate that part of the adsorbed Eu^3+^ is present as aquo ions, i.e. adsorbed as OS complexes. The other lifetime has a too large error for any reliable interpretation, but indicates complete or near-complete loss of hydration. A possible interpretation would be the incorporation of Eu^3+^ into biotite interlayers that may be accessible more easily here at the grain boundary. Such an interlayer exchange could occur as hydrated ions at frayed edges of the muscovite, with subsequent dehydration upon progression into the interlayer.

The determination of the sorption properties of biotite are complicated by its high Fe content, which leads to almost complete quenching of Eu^3+^ luminescence over wide areas on Bt and Bt*. Due to higher luminescence intensity, data could be obtained at areas **B** and **C**. EPMA shows a homogeneous distribution of Fe at areas **B** and **C**, so that decreased iron content cannot be responsible for the higher luminescence intensity in these areas. The homogeneous iron distribution on Bt and Bt*, also makes the presence of another mineral at the surface unlikely, as EPMA’s penetration depth is only about 3 µm. An even thinner film of another mineral would be expected to be removed entirely during polishing. The heterogeneity in Eu^3+^’s distribution can also be recognized in autoradiography results, where two biotite areas with different crystal orientations show a starkly different sorption behavior. We can assume that the same is the case in our µTRLFS measurements. Key for a high uptake capacity of Eu^3+^ by biotite is then uptake of Eu^3+^ into the interlayer of biotite by ion exchange. If the layers are parallel to the sample surface Eu^3+^ cannot enter the interlayers. Thus, the crystal orientation, strongly affects the amount of Eu^3+^ sorbed. Consequently, the Bt grain would have near-parallel surface layer orientation, while in the Bt* region the layers are more angled. A similar effect could also be related to other surface defects, such as cracks, which facilitate access to the interlayer, which could also be observed in areas with particularly strong ablation, when re-submerged in Eu^3+^ solution

## Conclusion

The results presented and discussed in the two previous sections highlight both, the capability of μTRLFS as a new analytical tool to elucidate sorption processes on the μm-scale, and the requirement to perform such an analysis for the improved understanding of sorption processes in natural rocks and other heterogeneous materials. From a technical perspective, relatively simple enhancements of the well-established TRLFS technique have enabled us to determine the speciation and sorption capacity of a natural rock within an ROI of 1 × 2 mm^2^ with a 20 μm grid. For each point, the full spectral information is obtained, i.e. not only luminescence intensity, but also band intensities and ratios, which enables the method to quantify sorption and determine the speciation responsible for the uptake. In addition lifetimes can be obtained, which give a direct measure of the luminescent probe’s hydration state. In principle these measurements could also be performed on every single point, measurement time considerations make select measurements at points of interest the better current option. Furthermore, these measurements are performed with trace amounts of adsorbed Eu^3+^, which could not even be detected by other microprobe techniques, synchrotron-μXRF and EPMA.

The results clearly show how differently the various minerals interact with Eu^3+^. While feldspar has a very high sorption capacity, bonding is relatively weak. On quartz the opposite is found, the capacity for sorption is very low, and may well have been disregarded for the sake of e.g. surface complexation or reactive transport modelling. However, the added information from the ^7^F_2_/^7^F_1_ spectral intensity ratio reveals that bonding is very strong, if it occurs. Immobilization of Eu^3+^ on quartz may then well be a significant retention process, as it would be expected to be most efficient at low concentrations. Another interesting observation is the distinct sorption properties of a mineral grain boundary, which could not have been determined in single phase, or even mixed powder studies. Even mineral grain orientation appears to have a significant effect on the uptake capacity as autoradiography demonstrates in the case of biotite. If the same applies to minerals, which have a less anisotropic, here layered, structure should be subject for additional research.

This novel technique should prove to develop into a valuable analytical tool for systems where heterogeneous behavior is expected or cannot be excluded, and thus help to reduce the experimental gap between well characterizable, but highly idealized model systems and highly relevant natural formations, where molecular level information is often unobtainable.

## Experimental Methods and Materials

### Sample preparation and characterization

The Eibenstock granite sample was obtained as a massive rock from a former uranium mine in Erzgebirge, Germany. A combination of powder X-ray diffraction (XRD) and X-ray fluorescence analysis (XFA) shows, that the Eibenstock granite mainly consists of quartz, K-feldspar, biotite and muscovite (Table 2). The minerals are not distributed homogeneously, with muscovite occurring as big shards and other typical grain sizes ~50 µm. The rock sample was processed in two ways: for batch experiments, a part of the rock was ground in an agate mill and sieved to grain sizes below 63 µm. For spatially resolved experiments, a part of the rock was cut into pieces of 23 × 23 × 10 mm³, glued onto glass slides with epoxy, ground down to a thickness of 150 µm, and finally polished. Polishing (Logitech polisher) was performed with a suspension of diamonds 1 µm and 3 µm in diameter, respectively, in ethanediol. The sample was washed with ethanol and deionized water (MilliQ, 18.2 MΩ), before the reaction with the Eu^3+^ solution.

**Table 2:** Mineral composition of Eibenstock granite.

Mineral	Vol. %
Quartz	45.0
K-feldspar	35.0
Na-feldspar	7.5
Biotite	7.5
Muscovite	4.0
Other opaque minerals	1.9

### Batch experiments

For batch experiments, the milled granite was immersed in a solution of 5 × 10^−5^ mol L^−1^ Eu^3+^ and 0.1 mol L^−1^ NaCl with a solid-to-liquid ratio (S/L) of 2 g/l. We prepared 30 samples with pH values in the range of 0.8 to 11.5. The pH values of each sample were adjusted daily over one week with HCl and NaOH. Afterwards the suspension was centrifuged for 30 minutes at 5300 rpm (≙ 6800 × g).

1 $${[E{u}^{3+}]}_{{\rm{sorbed}}}=(1-\frac{{[E{u}^{3+}]}_{{\rm{supernatant}}}}{{[E{u}^{3+}]}_{{\rm{blank}}}})\times 100 \% $$

The europium concentration of the supernatant was measured in triplicate by ICP-MS and the concentration of the europium stock solution was measured 12-fold. The percentage of sorbed Eu^3+^ was then calculated based on the measured concentrations. The error of [Eu^3+^]_sorbed_ was calculated using the statistical errors of the ICP-MS measurements and the error of pH measurement was set to ±0.1 pH units

### Spatially-resolved experiments

The thin-section sample was brought into contact with the Eu^3+^ solution in a custom-made PTFE flow cell (see Electronic Annex). The solution was pumped through the cell with a volumetric flow rate of 30 mL h^−1^ over the sample surface. The cross section of the flow cell at the sample was 28 mm². After the sorption experiment ended, remaining Eu^3+^ solution was flushed off the sample surface to prevent precipitation of Eu^3+^ solids. The same sample was used for microprobe X-ray fluorescence (µXRF), µTRLFS, electron probe microanalysis (EPMA), and autoradiography measurements.

For autoradiography measurements the thin-section was additionally set in a watch glass filled with 15 ml of a solution of the same solution, in which Eu^3+^ was replaced by ^152^Eu, a radioactive isotope of Eu that decays by electron capture or β^-^ decay with a half-life of 13.5 y. The sample side was faced to the solution to avoid sorption on the backside, which would produce background and distort the autoradiography image. After preparation, the sample was once more washed with deionized water.

A region of interest (ROI) on the thin-section sample was chosen by optical microscopy. The area should contain all three main mineral constituents: quartz, feldspar and biotite, which were identified visually.

### µXRF

The µXRF measurements were conducted at the INE-Beamline at the KIT Synchrotron light source in Karlsruhe, Germany^52^. The X-ray photon beam had an incident energy of 18 keV, well above the K- or L-edges of the mineral constituents as well as Eu^3+^. The incident photon flux was tracked by an ionization chamber to normalize the fluorescence intensity maps. The beam was focused to a spot of approximately 25 µm (FWHM) in diameter using polycapillary half-lenses and the sample was scanned in a 10 µm grid. Because the measurement was performed in air, no elements with atomic numbers below that of argon (Z = 18) were detectable. The spectral resolution

2 $$\varepsilon =FWHM/E$$

of the Hitachi Vortex VX60 SDD detector under the experimental conditions (high count rate, line purity not always given) was determined to be (3.3 ± 0.1)% by evaluating the X-ray fluorescence peaks between 6.4 keV and 14.9 keV. The determined spectral resolution is slightly worse than that of a Fe calibrating source (FWHM of the Mn K fluorescence line is 140 eV – 150 eV), which give 2.5%. Consequently, fluorescence peaks will overlap if their energy is too close to each other, which hinders the unambiguous identification of some elements. In this case this is of importance for the identification of Eu (*Lα*_1_ = 5,849 keV, *Lβ*_1_ = 6,458 keV), which overlaps with the common impurity Mn (*Kα*_1_ = 5,900 keV, *Kβ*_1_ = 6,492 keV) because their peaks FWHM (*FWHM*([*K*/*L*]*α*_1_) ≈ 0.19 keV, *FWHM*([*K*/*L*]*β*_1_) ≈ 0.21 keV) is much higher than the distance between Eu and Mn fluorescence peaks (Δ[*K*/*L*]*α*_1_ = 0.051 keV, (Δ[*K*/*L*]*β*_1_ = 0.034 keV). So if both elements are present in one location it is not possible to distinguish them, especially if one of the elements is present only in trace concentrations and the signal strength is low. Neither the L-shell transitions of Mn (~640 eV) nor the Eu K-shell transitions could be used for distinguishing the elements, as the beam energy was insufficient to reach Eu^3+^’s K-edge and attenuation in air becomes prohibitive at the low energy of Mn L-edges.

### µTRLFS

**Figure 5:**
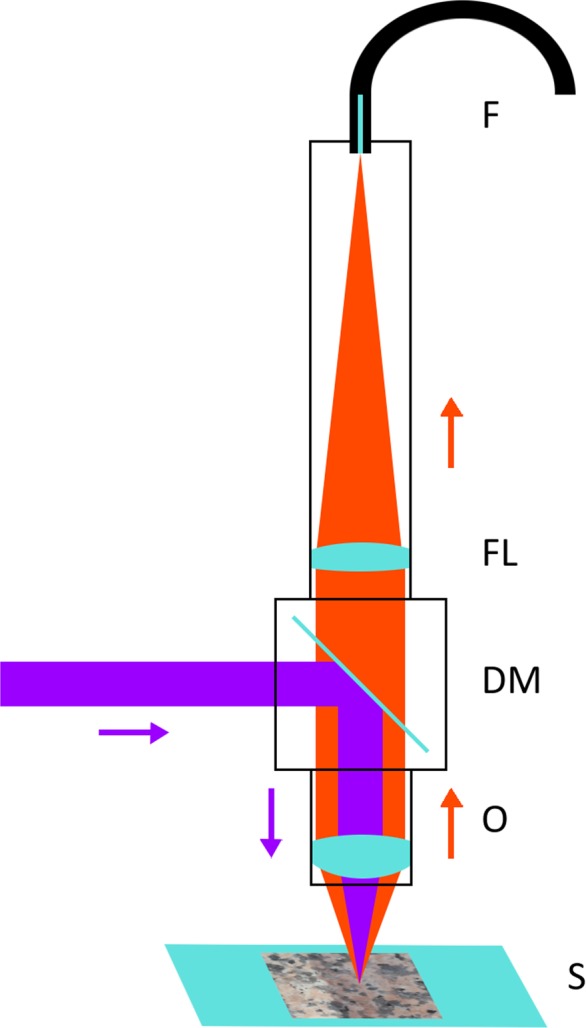
Scheme of the µTRLFS setup with dichroic mirror (DM), objective for focusing and collimating (O), sample on XYZ-stage (S), focusing lens for the luminescence light (FL), fiber connected to spectrometer (F), UV-laser for excitation (purple) and emitted luminescence light (orange).

µTRLFS measurements were performed with a specially designed setup (Fig. 5). A laser beam (Surelite SL I-20 @355 nm pump laser, Continuum with NarrowScanK @Exalite 389/398 mix dye laser, Radiant Dyes) was coupled into the setup by a dichroic mirror (490 nm cut-off wavelength, Thorlabs). The redirected beam was then focused onto the sample by an objective (HCX APO 10×, Leica). The sample was placed on an XYZ motorized stage (Newport), which is used for moving the sample surface into the focal plane (Z) and scanning the sample through the focal point (XY). The incident light resonantly excites Eu^3+^ in the focal point and the emitted luminescence is collected and collimated by the focusing objective. The fluorescence light passes the dichroic mirror and is focused by a second lens (achromatic, f = 100 mm, Thorlabs) onto the tip of a light guide. The light guide was connected to a spectrometer (Shamrock SR303i spectrograph with DH320T-18U-63 iCCD camera, Andor, UK) for detection. By scanning the sample surface a pixelated map with full spectral information in each point can be obtained.

The laser beam was focused to ~20–30 µm in diameter and a 20 µm scanning grid was chosen. The excitation wavelength was set to 394 nm, the resonant wavelength of the ^7^F_0_ → ^5^L_6_ transition of Eu^3+^, which shows the strongest absorption and is thus most sensitive^28^. The pulse energy was set to ~50 µJ by two crossable polarizers (Glan-Taylor polarizer, Thorlabs). This low excitation energy was intentionally chosen to minimize any destructive effects of the focused laser beam. The signal was accumulated 200 times in each point resulting in a time of 10 seconds needed for each point. The measurement of the whole map (1836 data points) needed approximately six hours including time for moving the sample.

µTRLFS spectra were evaluated by a Python script to generate images of the total fluorescence by integrating the emission bands of the ${}^{5}D_{0}\to {}^{7}F_{1}$ and ${}^{5}D_{0}\to {}^{7}F_{2}$ transition to get information about the sorption capacity and the fluorescence ratio by dividing the ${F}_{2}$ and ${F}_{1}$ emission bands to get information about the speciation. The background of the spectra was approximated to be linear and was determined based on both edges of an emission band. After the mapping, lifetimes were measured in selected spots. Spectra for lifetime analysis were background corrected by the same method. The luminescence decay profiles were reproduced with mono- or biexponential decay curves to obtain the luminescence lifetime τ. The lifetime can then be correlated to the number of coordinating water molecules N(H_2_O) by the empirical Horrocks equation^53,54^:

3 $$N({{\rm{H}}}_{2}{\rm{O}})\pm 0.5=\frac{1.07\,\mathrm{ms}}{\tau [{\rm{ms}}]}-0.62$$

### Autoradiography

For imaging the spatial distribution of the ^152^Eu sorption, the granite thin-section was covered with a thin plastic foil, to avoid contamination, and placed onto a BAS-IP MS imaging plate (GE Lifesciences). The plate was exposed for up to 60 min and the image was read out with a spatial resolution of 10 µm using an Amersham Typhoon biomolecular imager. The actual spatial resolution of the obtained autoradiography images is lower than the theoretical value, because of the isotropic emission of radiation from each point.

### EPMA

EPMA measurements were conducted at the Helmholtz-Institute Freiberg for Resource Technology with a JEOL JXA 8530 F (JEOL Ltd, Tokio, Japan). The setup consists of a field emission electron gun, five wavelength dispersive spectrometers (WDS), which are equipped with different analyzer crystals and an energy dispersive spectrometer (EDS). The sample was scanned in a 4 µm grid with a beam diameter of 3 µm with an acceleration voltage of 20 kV and a dwell time of 200 ms. Element distribution mappings were recorded for V (PETL analyzer), Mn (LIFH analyzer), Y (TAP analyzer) and Eu (LIFH analyzer) with the WDS. EDS was used to get the elemental distribution of the main matrix elements Na, Mg, Al, Si, P, K, Ca, and Fe. By using WDS detection for Eu and Mn it is possible to distinguish between both elements even though their fluorescence energy is close to each other.

## Supplementary information

Supplementary Information

## Data Availability

The datasets generated during and/or analysed during the current study are available from the corresponding author on reasonable request.
