# Supplementary material for: Sorption of Eu(III) on Eibenstock granite studied by µTRLFS: A novel spatially-resolved luminescence-spectroscopic technique
Source: Sci Rep. 2019 Apr 18;9:6287. doi: 10.1038/s41598-019-42664-2 (PMC6472502; doi:10.1038/s41598-019-42664-2)
Supplement: Supplementary file 1 — Supplementary Information [file 41598_2019_42664_MOESM1_ESM.pdf]

Supplementary Information

Sorption of Eu(III) on Eibenstock granite studied by μTRLFS: A novel spatially-resolved luminescence-spectroscopic technique

K. Molodtsov,[a] S. Schymura,[a] J. Rothe,[b] K. Dardenne,[b] M. Schmidt[a,*]

[a] *Helmholtz-Zentrum Dresden-Rossendorf, Institute of Resource Ecology, Dresden, Germany*

[b] *Karlsruhe Institute of Technology (KIT), Institute for Nuclear Waste Disposal (INE), Karlsruhe, Germany*

* *Corresponding author (moritz.schmidt@hzdr.de)*

A Quantitative Eu^{3+} distribution (µXRF, EPMA)

In Supplementary Figure A.1 the distribution for Mn and Eu measured with µXRF is shown. Due to signal overlap, it cannot be distinguished between Eu and Mn. The µXRF data only shows Eu+Mn signals in the Bt and Bt* region and is spread relatively homogeneous. To figure out which of both elements is responsible for the shown distribution another experiment was conducted. An additional sample was measured prior and after sorption of Eu^{3+} under the same conditions, but no difference in the Eu/Mn distribution could be determined. Apparently, the sensitivity of this method is not high enough to detect Eu^{3+} sorption on a granite surface with the chosen experimental conditions. Because of this, the shown Mn+Eu distribution most likely corresponds mainly to Mn, which is in a good agreement with the coincident distribution of Mn+Eu and Fe due to Fe substitution by Mn. The EPMA measurement also shows that the Mn distribution is identical to the Mn + Eu distribution of the µXRF measurement.

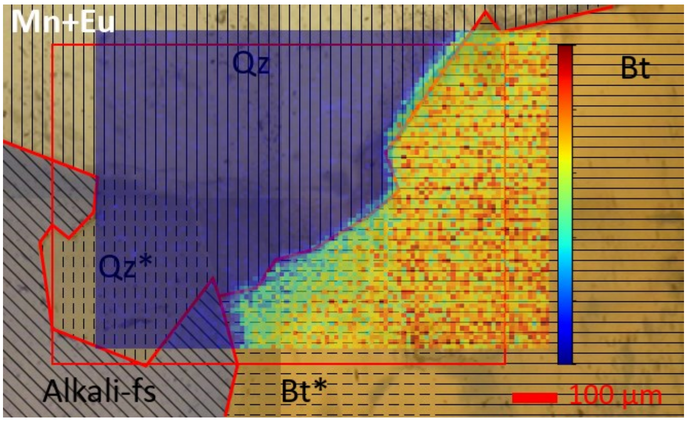

Supplementary Figure A.1: Elemental distribution measured with µXRF of the overlapping manganese and europium signal with a red box indicating the ROI chosen for µTRLFS measurements

With EPMA the Eu distribution was measured by WDX which has a higher sensitivity and energy resolution than EDX to be able to distinguish between Eu and Mn. Apparently the sensitivity of this method was also not sufficient to detect the europium sorption complexes with the chosen experimental conditions as there is no Eu detectable on the ROI (Supplementary Figure A.2).

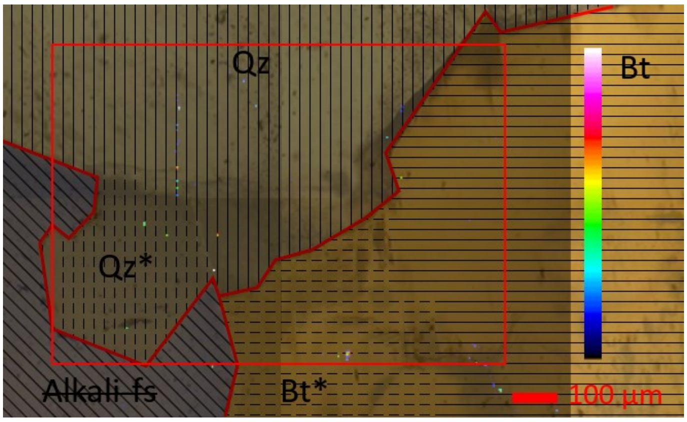

Supplementary Figure A.2: Elemental distribution of europium measured with EPMA with a red box indicating the ROI chosen for µTRLFS measurements

In Supplementary Figure A.3 the results of the autoradiography measurement are shown. The sample preparation and measurement were conducted after the µTRLFS measurement so that the surface on the ROI was already altered by laser ablation of the focused beam regarding its roughness. Because of this more Eu^{3+} was sorbed at the ROI, especially on biotite in comparison to the previous measurements. To avoid this influence we characterized surrounding areas with the same mineralogy.

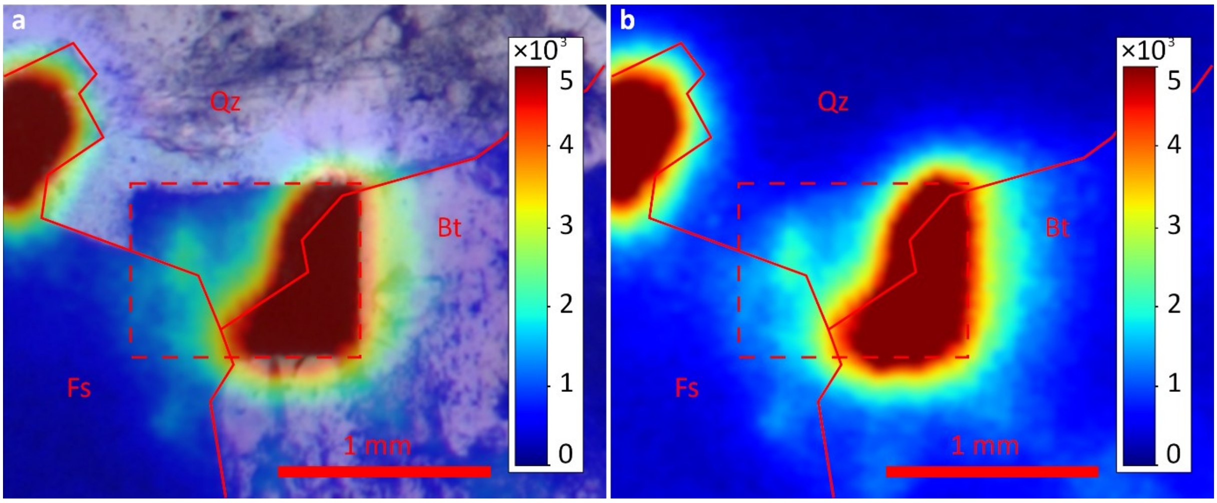

Supplementary Figure A.3: Autoradiography image of the ROI with underlying image of thin-section microscopy (**a**) and standalone (**b**) with red outlines of each mineral phase at the surface and red dotted box indicating the ROI measured with µTRLFS

The surrounding quartz area shows the lowest counts of ^{152}Eu with a homogeneous distribution. On feldspar the counts area higher and also show a slight inhomogeneity to the right and a hot spot to the upper edge. Biotite seems to have a similar intensity as feldspar and also shows an inhomogeneous distribution.

B Sample cell

In Supplementary Figure B.1 the flow cell, which was used for sample preparation is shown. It is nearly completely made of PTFE, except for the inlet and outlet tubes (silicone), sealings (rubber) and the window above the sample holder (borosilicate glass). The gap between the window and the sample is adjustable to simulate different flow patterns. The window serves as a preparation for in-situ measurements directly in the cell. For sample preparation the gap was set to 1 mm and the Eu^{3+} solution was pumped out of a reservoir through the inlet tube over the sample and then through the outlet tube into a waste reservoir.

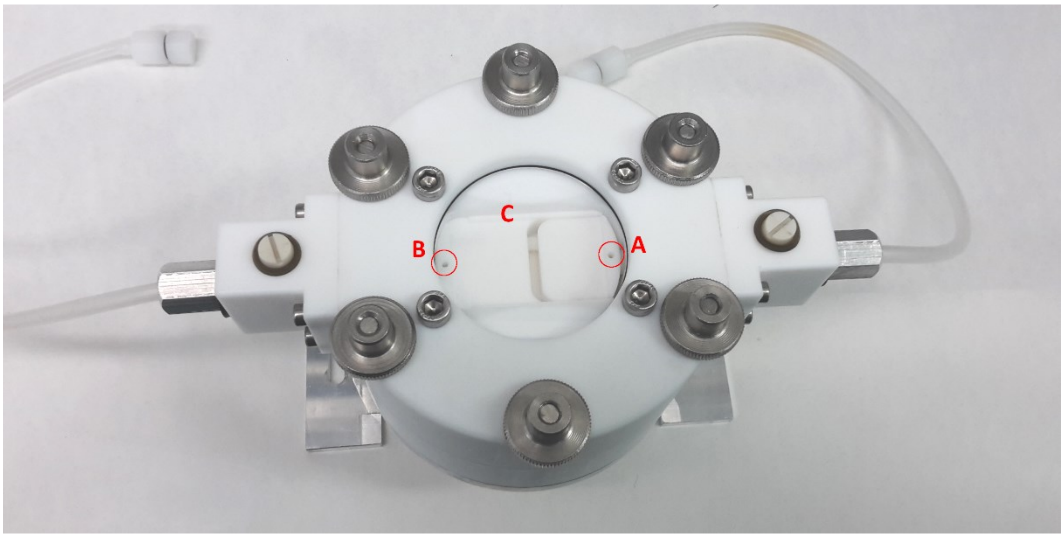

Supplementary Figure B.1: PTFE flow cell with inlet (A), outlet (B) and sample holder (C)

C µXRF spectra

In Supplementary Figure C.1 XRF spectra are shown as an example for each mineral, that was identified within the ROI. In each spectrum, arrows point to the energies of the fluorescence lines of elements that were shown as a distribution within this publication. There are additional lines, that were identified as arsenic (10.5 keV / 11.7 keV), zinc (8.6 keV / 9.6 keV) and copper (8.0 keV / 8.9 keV). The lines of titanium (4.5 keV / 4.9 keV), barium (4.5 keV / 4.8 keV) and lanthanum (4.6 keV / 5.0 keV) are overlapping and can therefore not be distinguished. The spectra are normalized to the incident photon flux and are quantitative results.

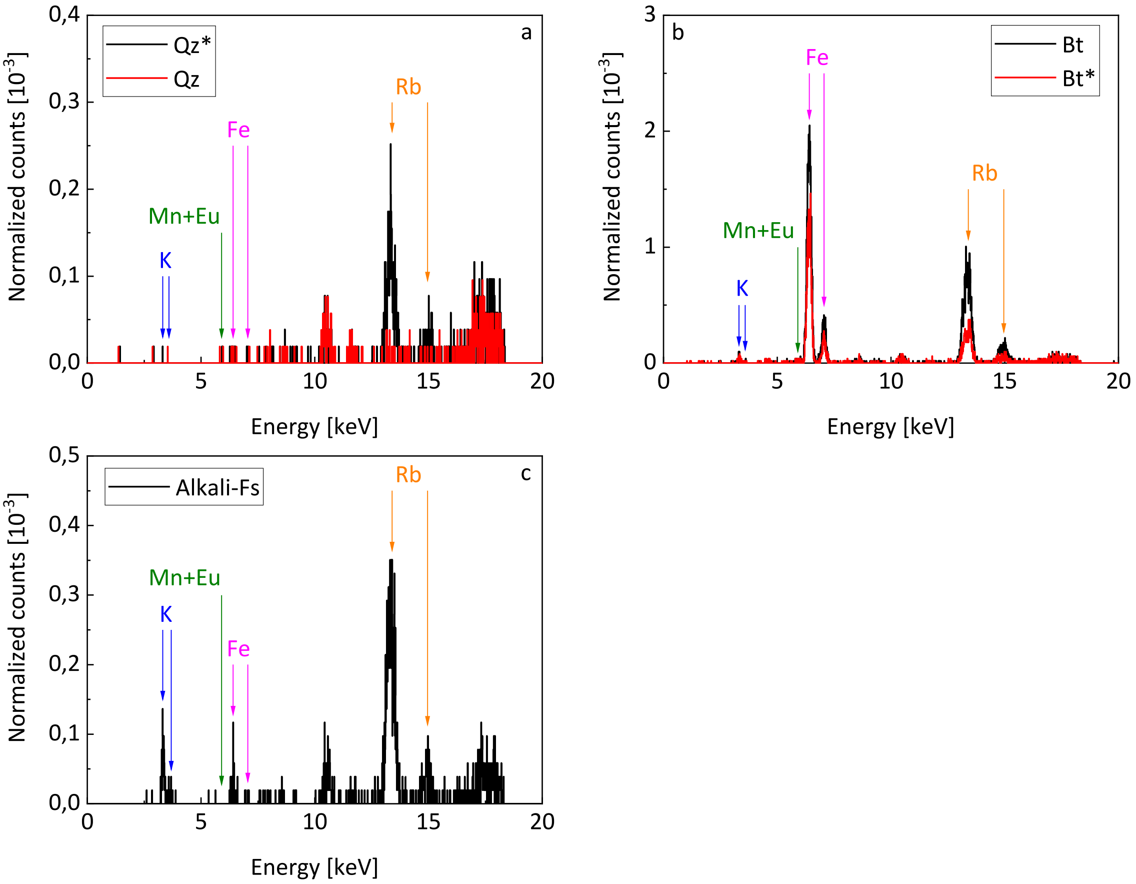

Supplementary Figure C.1: Normalized XRF spectra (regarding the incident photon flux) as an example for Qz and Qz* (a), Bt and Bt* (b) and Alkali-Fs (c) with indicators for elements that were shown as a distribution
